# Sinus laser-assisted closure (SiLaC) in pilonidal sinus disease: a multicenter cohort study

**DOI:** 10.1007/s10151-025-03280-7

**Published:** 2026-05-13

**Authors:** J. de Kort, L. V. van Kempen, R. Schouten, H. Brokx, R. Erkens, A. M. I. Amir, M. R. Vriens, E. F. A. Smakman, N. Smakman, E. J. B. Furnee

**Affiliations:** 1 Department of Surgery, Diakonessenhuis, P.O. Box 80250, 3508 TG Utrecht, The Netherlands; 2 Department of Surgery, Flevoziekenhuis, Almere, The Netherlands; 3 Department of Surgery, Bravis Ziekenhuis Bergen Op Zoom, Roosendaal, The Netherlands; 4 Department of Surgery, Treant Hospital, Emmen, The Netherlands; 5 Department of Surgery, University Medical Center Utrecht, Utrecht, The Netherlands; 6 Department of Abdominal Surgery, University Medical Centre Groningen, Groningen, The Netherlands

**Keywords:** Pilonidal sinus, Laser ablation, Retrospective study

## Abstract

**Background:**

Sinus laser-assisted closure (SiLaC) is gaining popularity as a minimally invasive surgical technique to treat chronic sacrococcygeal pilonidal sinus disease (SPSD). However, long-term outcomes on this technique are rarely reported in the literature. This multicenter study aimed to report the mid-term outcome of a cohort of patients who underwent SiLaC treatment for primary or recurrent SPSD.

**Methods:**

Patients with primary or recurrent SPSD were included in this retrospective cohort study. Data were collected from medical records, and a validated questionnaire was distributed to all patients at follow-up to assess symptoms, quality of life, and the need for additional surgery.

**Results:**

A total of 231 patients were included, with a median follow-up of 33 months (IQR 20–44): 128 patients underwent SiLaC for primary SPSD and 103 for recurrent SPSD. Additional interventions were required in 35 patients with primary SPSD (27.3%) and in 30 patients with recurrent SPSD (29.1%). Repeat SiLaC alone, i.e. no other surgical treatment than repeat SiLaC, was performed in 25 patients (19.6%) in the primary group and 22 patients (21.4%) in the recurrent group. Treatment failure, defined as persistent symptoms and/or the need for surgery other than SiLaC, occurred in 23 patients (18.0%) in the primary group and in 21 patients (20.4%) in the recurrent group. However, SPSD-related symptoms were low at follow-up, and quality of life measured by the SF-36 was reported to be even higher than in the general USA population, both in the primary and recurrent group.

**Conclusions:**

SiLaC for primary and recurrent SPSD appeared to be a safe procedure with excellent symptom control and quality of life, with only 7.8% of patients in both groups requiring other surgical treatment than SiLaC.

## Introduction

Minimally invasive surgical techniques have gained more and more popularity over radical excision in the treatment of chronic sacrococcygeal pilonidal sinus disease (SPSD) over the recent years as these techniques offer several advantages, including smaller postoperative wounds, faster return to normal daily activities, less pain, and the ability to perform some procedures in an outpatient setting. Different minimally invasive techniques are available, including pit picking [1, 2], phenolization of the sinus tract, endoscopic treatment and sinus laser assisted closure (SiLaC). The latter technique was introduced in 2011 [3] after its successful application in the treatment of peri-anal fistulas [4] and is applied more and more commonly as primary treatment of chronic SPSD.

Several studies on SiLaC with short-term follow-up have shown favorable outcomes after one or more SiLaC treatments for chronic SPSD, with recurrence rates ranging from 2.9% to 15.2% [5–10]. Also, two systematic reviews on SiLaC have been performed reporting recurrence rates between 3.8% and 7.6% after a follow-up of 12 months [11, 12]. To date, however, the literature predominantly describes short- to mid-term results after SiLaC with follow-up times up to 24 months [13], and only one retrospective cohort study with long-term follow-up has been performed, showing a recurrence rate of 14.5% with a follow-up of 5.2 years in 83 patients after SiLaC [14]. However, this was a single-center study only focusing on recurrence rate, whereas the long-term symptomatic outcome and quality of life were not reported in this study.

The aim of this multicenter study was to report the mid-term outcome of a cohort of patients who underwent SiLaC treatment for primary or recurrent SPSD, focusing on symptomatic outcome, quality of life, and the need for additional surgery.

## Methods

This multicenter retrospective cohort study included patients aged over 18 years who underwent SiLaC for the treatment of SPSD between June 2019 and June 2024. Patients were included from three Dutch hospitals: Treant hospital Emmen, Flevoziekenhuis Almere, and Bravis hospital Bergen op Zoom/Roosendaal. Patients with a pilonidal abscess or with the suspicion of an extensive network of subcutaneous tracts were excluded, as these patients were treated differently. Patients were analyzed in two different groups, depending on whether SiLaC was performed for primary SPSD (primary group) or for recurrent SPSD (recurrent group). The Medical Ethics Committee in Utrecht, the Netherlands, approved this study.

### Surgical technique: procedural steps of the SiLaC treatment

**Fig. 1:**
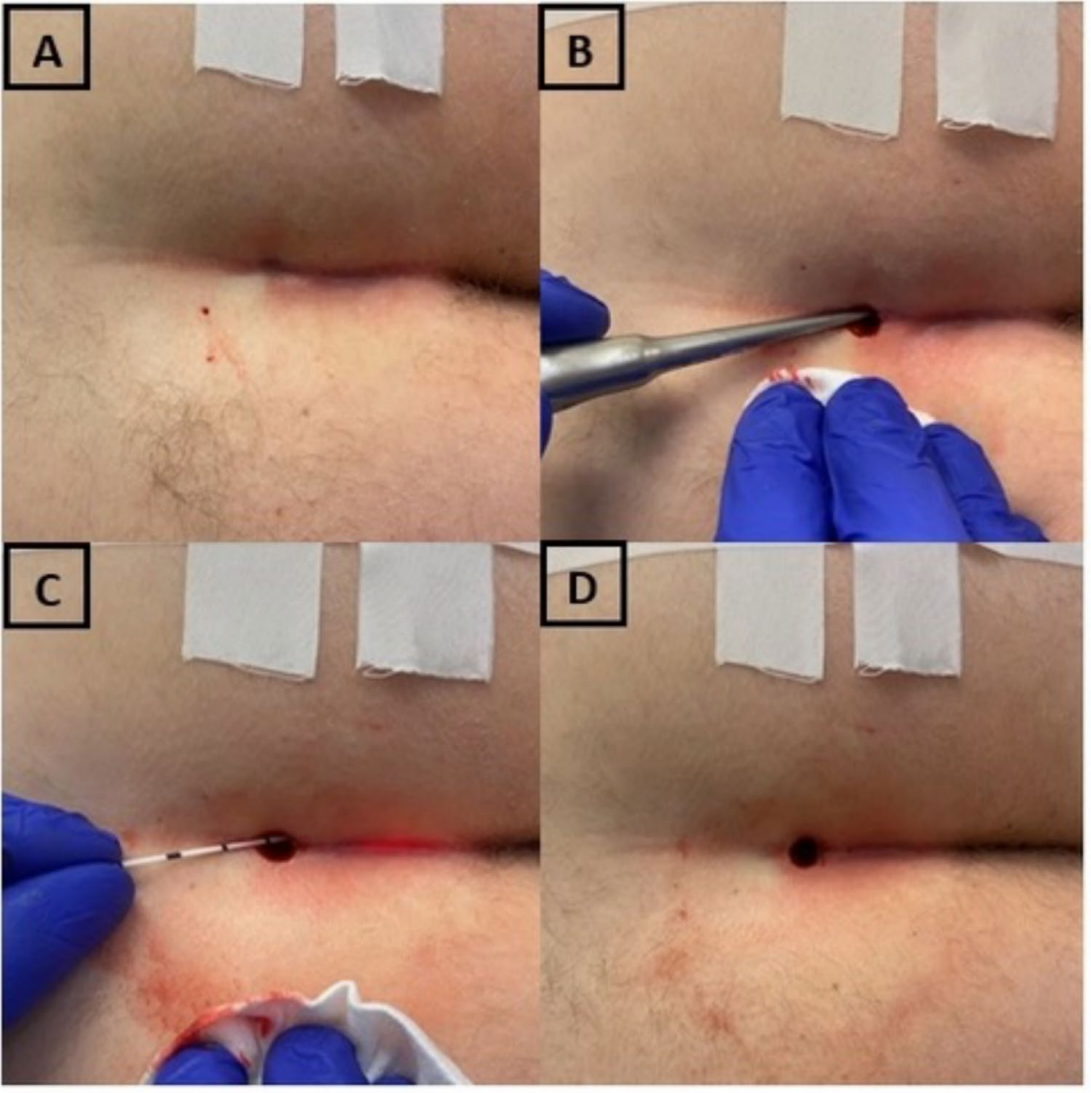
Procedural steps of the sinus laser-assisted closure (SiLaC) technique. A: the buttocks are taped to the operating table for an adequate view of the sacrococcygeal area. The area is shaved. B: the sinus pit is probed and the tract is cleaned of hair and debris with a curette. C: a radial laser probe is fully inserted and subsequently retracted with 1 mm per second. D: the wound is left open after SiLaC

SiLaC treatment was performed either in the operating theater or the outpatient department, depending on the patients’ and physicians’ preferences. Local, spinal, or general anesthesia was administered as appropriate. The patient was positioned sideways and the buttocks were taped to the side of the table to create an optimal view of the sacrococcygeal area, which was then shaved using a surgical knife or an electric razor (Fig. 1).

First, the most cranial sinus pit was probed to identify the position of the underlying tract. Following identification, a curette was used to clean the tract(s) of hair and debris, and saline solution was used to flush the tract. If necessary, more sinus pits were probed to clean the complete tract. Then, the laser probe was inserted, and when fully inserted, the probe was activated and withdrawn at a continuous rate of 1 mm per second. The 360-degree energy pulses emitted by the probe destroys the epithelium, causing the tract to shrink and close. In addition, any residual hair and debris adheres to the probe as it is retracted from the sinus tract. If the tract did not fully close, the probe was reinserted for a second application. The amount of joules administered depended on the extensiveness of the tract(s). After the procedure, a gauze was applied to the sacrococcygeal region. Patients were scheduled for follow-up appointments until full wound closure was achieved.

### Data collection

Following identification of all patients who underwent SiLaC in the three hospitals during the given period, data were retrospectively collected from the medical notes. Baseline characteristics included age, sex, weight, height, smoking status, family history of SPSD, and time spent in a sitting position. Intraoperative observations (procedure duration, number of sinus pits, tract length, total joules) were extracted from medical records. Previous surgical interventions in the recurrent group, as well as postoperative complications (bleeding, readmission, other adverse events), were also recorded.

To obtain mid-term outcome, a questionnaire was emailed to all included patients. Quality of life was obtained by the Short Form (SF)-36 [15] and compared with the general USA population [15]. In addition, patients were asked about their current status of the disease (on a 4-point scale: completely healed, better but not completely healed, unchanged, worse), and their current general health status (on a 3-point scale: improved, unchanged, worse), both compared with preoperatively. In addition, patients were asked whether they found the treatment to be beneficial, whether they would undergo the same treatment again, and also to rate the overall burden of their treatment for SPSD, the latter one on a visual analogue scale (VAS)-score, with a lower score indicating a lower burden of treatment.

Symptoms related to SPSD were evaluated on a 6-point Likert scale (0 indicating no complaints, 5 indicating daily complaints). The most prevalent symptoms related to SPSD were assessed: fluid discharge, pain, itching, irritation, and burning sensation at the natal cleft.

Finally, medical records were examined for additional interventions and patients where asked in the questionnaire whether they had undergone further surgical treatment after SiLaC in another hospital, including the number and type of procedures performed, in order to collect data on any further surgical treatments after the primary SiLaC treatment. Patients were also asked if they had the impression of persistent or recurrent SPSD; if they responded affirmatively, they were invited for an appointment at the outpatient clinic and treated according to the shared decision-making principle. In addition, the questionnaire inquired whether patients are regularly epilating the natal cleft.

### Statistical analysis

All collected data were analyzed using SPSS for Windows version 29.0 (SPSS Inc., Chicago, IL, USA). Values were expressed as mean (standard deviation, SD) or median (interquartile range, IQR), depending on whether data were normally distributed or not, respectively. For statistical analysis of dichotomous and categorical values, the Pearson Chi square test was used. A one-sample *t*-test was conducted to compare mean scores of the obtained data with a reference mean of the general population. Significance was set at *p* = 0.05.

## Results

A total of 231 of the 754 approached patients from the three different hospitals were included in the current study during the inclusion period. SiLaC was performed as primary intervention in 128 patients (55.4%) and in the remaining 103 patients (44.6%) SiLaC was performed for recurrent SPSD as these patients underwent a previous surgical treatment for SPSD. Baseline characteristics of the cohort of patients who underwent SiLaC for primary or for recurrent SPSD are presented separately in Table 1.

**Table 1:** Baseline characteristics and intraoperative data

	Primary SPSD (*n* = 128)	Recurrent SPSD (*n* = 103)
Male sex (%)	111 (86.7)	66 (64.1)
Age (years)	28 (25–38)	29 (24–38)
Body mass index (kg/m^2^)	26.0 (23.6–28.5)	26.9 (24.6–30.4)
Smoking (%)	33 (25.8)	27 (26.2)
Number of cigarettes	10 (5–15)	14 (6–19)
Family history of SPSD (%)	23 (18.0)	23 (22.3)
Seated position (h/day)	8 (5–9)	6 (5–8)
Duration of follow-up (months)	31.5 (19.3–41.8)	34.0 (21.0–47.0)
Intraoperative data		
Operative time (minutes)	10 (7–14)	10 (7–14)
Sinus pits at surgery (n)	2 (1–3)	2 (1–3)
Length of sinus tract (centimeters)	5 (3–6)	6 (4–8)
Energy administered (joules)	697 (452–987)	801 (518–1367)

SPSD, sacrococcygeal pilonidal sinus disease

Values are reported as median (interquartile range, IQR)

In the recurrent group, 51 patients (49.5%) were treated with incision and drainage prior to their initial treatment with SiLaC. In addition, 52 patients (50.5%) underwent another surgical intervention prior to the initial SiLaC treatment, such as excision with primary or secondary wound healing (*n* = 39), phenolization of the sinus tract (*n* = 9), excision with VY-flap reconstructions (*n* = 1), SiLaC in another hospital (*n* = 2), or a combination of procedures (*n* = 1).

Intraoperative data in the primary and recurrent SiLaC group are presented in Table 1. Postoperative bleeding was reported in one patient in either group. In addition, one patient in the recurrent group was readmitted due to postoperative fever, which was conservatively treated. No other postoperative complications occurred in either group.

### Quality of life and subjective outcome

**Fig. 2:**
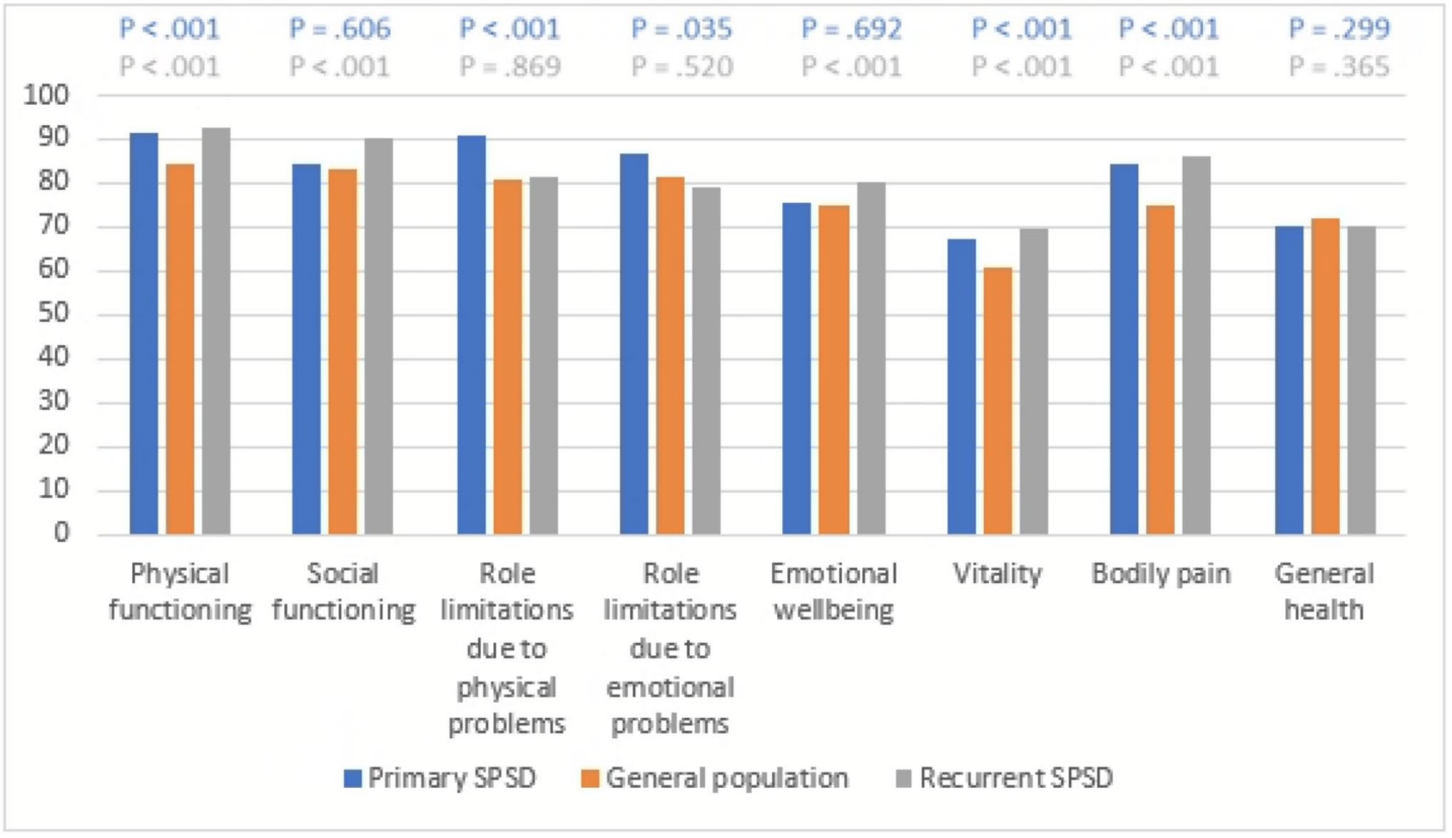
Short Form 36 (SF-36) at follow-up after SiLaC for primary and recurrent sacrococcygeal pilonidal sinus disease compared with the general population [16]. *P*-values expresses association between either group and the general population

Quality of life, as assessed using the SF-36, was scored significantly higher in several domains in both groups compared with the general USA population [16] (Fig. 2).

The primary group scored significantly higher than the general USA population in the domains physical functioning, role limitations due to physical problems, role limitations due to emotional problems, vitality, and bodily pain. The recurrent group scored significantly higher than the general USA population in the domains physical functioning, social functioning, emotional well-being, vitality, and bodily pain. In the domain role limitations due to emotional problems, only patients in the recurrent group scored slightly lower compared with the general USA population (79 versus 81.3), while both groups scored a bit lower in the general health domain: mean score of 70.3 for both treatment groups compared with 72 for the general USA population [16]. These differences, however, were not significantly different.

SPSD-related symptoms at follow-up were absent or only minimal in both groups (Table 2). General health compared with preoperatively, the disease status compared with preoperatively as well as the perceived benefit of the treatment were all positively rated, although only about 60% of patients in both groups opted for the highest scores in these categories. Nevertheless, the majority of patients in the primary group (*n* = 105, 82.0%) and the recurrent group (*n* = 82, 79.6%) would choose the same treatment again. The burden of the treatment was rated as approximately 40 out of 100 in both groups (Table 2).

**Table 2:** Subjective outcome

	Primary SPSD (*n* = 128)	Recurrent SPSD (*n* = 103)
Symptoms at natal cleft^a^		
Fluid discharge	0.0 [0.0–1.0]	0.0 [0.0–1.0]
Itch	0.0 [0.0–1.0]	0.0 [0.0–1.0]
Pain	0.0 [0.0–1.0]	0.0 [0.0–1.0]
Irritation	0.0 [0.0–1.0]	0.0 [0.0–1.0]
Burning sensation	0.0 [0.0–0.0]	0.0 [0.0–0.0]
General health compared with preoperatively		
Better (%)	68 (53.1)	61 (59.2)
The same (%)	54 (42.2)	37 (35.9)
Worse (%)	6 (4.7)	5 (4.9)
Status of disease compared with preoperatively		
Completely healed (%)	77 (60.2)	67 (65.0)
Better, but not completely healed (%)	34 (26.6)	23 (22.3)
The same (%)	14 (10.9)	11 (10.7)
Worse (%)	3 (2.3)	2 (1.9)
Treatment considered beneficial		
Very much (%)	80 (62.5)	62 (60.2)
Quite a bit (%)	26 (20.3)	17 (16.5)
A little bit (%)	14 (10.9)	12 (11.7)
Not at all (%)	8 (6.3)	12 (11.7)
Choosing the same treatment again		
Yes (%)	105 (82.0)	82 (79.6)
Indifferent (%)	14 (10.9)	13 (12.6)
No (%)	9 (7.0)	8 (7.8)
Burden of treatment (VAS-score, 0–100)	41.5 [20.0–61.5]	40.0 [20.0–65.0]

SPSD, sacrococcygeal pilonidal sinus disease; VAS, visual analogue scale

Values are reported as median and interquartile range (IQR) unless otherwise stated

^a^Items scored on a 6-point scale (0 meaning no complaints, 5 meaning daily complaints)

### Additional interventions after SiLaC

Following the initial SiLaC treatment, in 93 patients (72.7%) in the primary group and in 73 patients (70.9%) in the recurrent group, no additional intervention was necessary during the follow-up period. However, the remaining 65 patients (35 patients in the primary group, 27.3%, and 30 patients in the recurrent group, 29.1%) underwent at least one subsequent surgical intervention after initial SiLaC. Median time between the initial SiLaC treatment and the secondary treatment was, regardless of type, 7 months (IQR 5–12) for the primary group and 6 months (IQR 3.8–12) for the recurrent group. Median time between secondary treatment and third treatment was 14 months (IQR 12–23.3) and 18 months (IQR 10.8–27.8), respectively. The overall median follow-up of the subgroup of patients who received more than one intervention was 36 months (IQR 27–45) for the primary group (*n* = 35) and 36.5 months (IQR 23.5–55) for the recurrent group (*n* = 30).

**Fig. 3:**
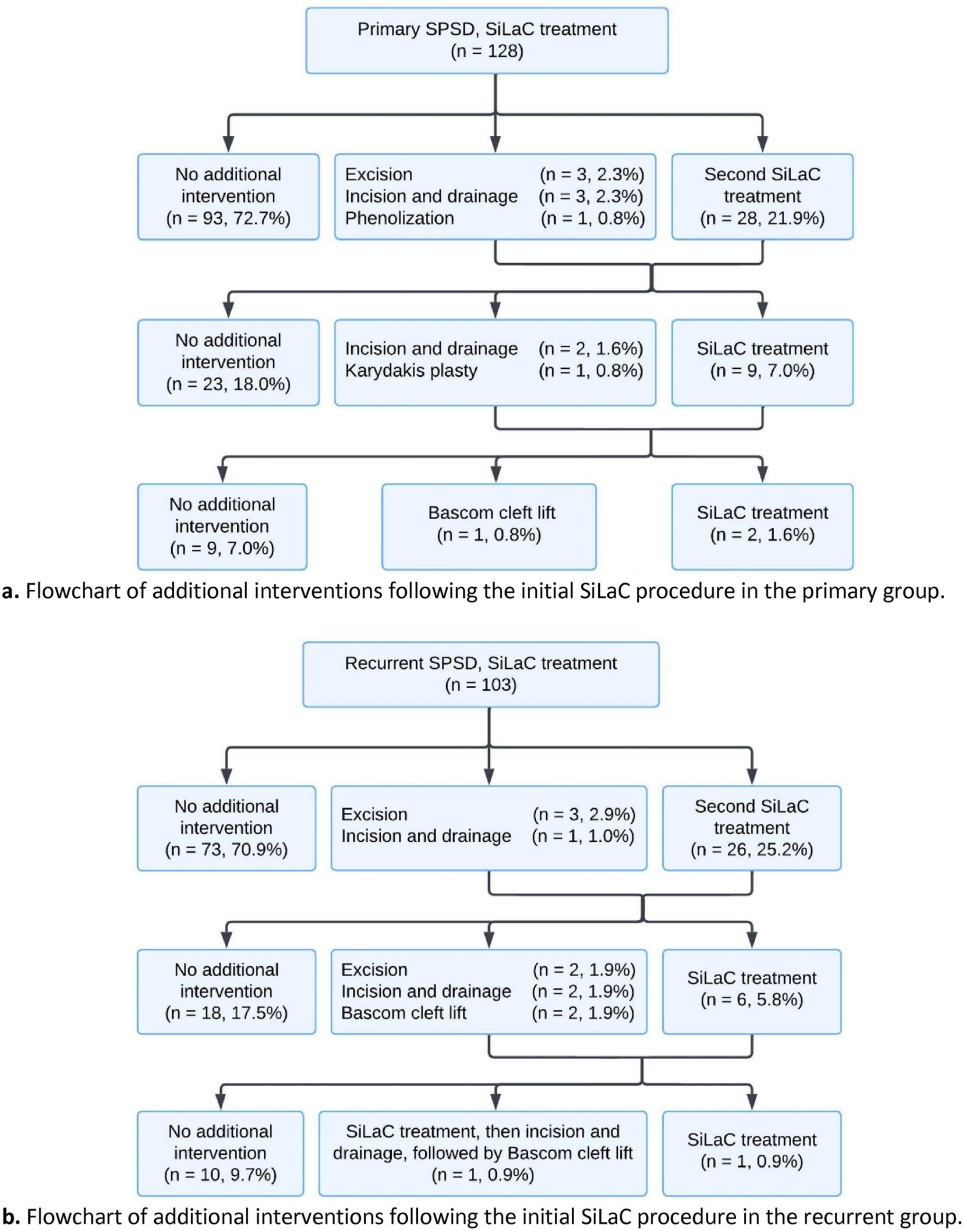
**a** Flowchart of additional interventions following the initial SiLaC procedure in the primary group. **b** Flowchart of additional interventions following the initial SiLaC procedure in the recurrent group

Most additional interventions (*n* = 54, 83.1%) involved another SiLaC treatment: in 28 patients (21.9%) in the primary group (Fig. 3a) and in 26 patients (25.2%) in the recurrent group (Fig. 3b). In the primary group, seven patients (5.4%) underwent another type of surgery following the initial SiLaC (Fig. 3a). In addition to a third SiLaC procedure in nine patients (7.0%), another surgical intervention was performed as tertiary treatment in three patients (2.3%) in the primary group. In the recurrent group, a different type of surgical treatment was performed in four patients (3.9%) after initial SiLaC (Fig. 3b). Alongside a third SiLaC procedure in six patients (5.8%), another surgical intervention was performed as tertiary treatment in six patients (5.7%) in the recurrent group. A fourth surgical procedure was necessary in three patients in the primary group (Fig. 3a) and in two patients in the recurrent group (Fig. 3b).

At follow-up, 15 patients (11.7%) in the primary and 13 patients (12.6%) in the recurrent group indicated in the questionnaire that they still were experiencing persistent SPSD. All those patients were contacted and upon contact, five patients in either group stated that they were either too busy or that their symptoms were not bothersome enough to make an appointment. Four patients in the primary group (3.1%) and seven patients in the recurrent group (6.8%) were scheduled for an appointment and are awaiting treatment (*n* = 2 in either group) or have had treatment (primary group: phenolization, *n* = 1, excision with secondary healing, *n* = 1; recurrent group: conservatively treated, *n* = 2, Bascom cleft lift, *n* = 1, re-SiLaC, *n* = 1, incision and drainage followed by re-SiLaC, *n* = 1). Lastly, six patients in the primary group and one patient in the recurrent group could not be contacted despite repeated attempts. In total, treatment failure, i.e. persistent symptoms and/or the need for a different type of surgical treatment (other than SiLaC), was observed in 23 patients (18.0%) in the primary group and in 21 patients (20.4%) in the recurrent group.

At follow-up, 82 patients (35.5%) indicated keeping their natal cleft hair-free: 35 using laser hair removal, 26 with depilatory cream, 18 with a razor, and 3 through epilation. Hair removal, regardless of the method used, was not associated with the need for additional surgery (*p* = 0.330).

## Discussion

This study reports the outcomes of SiLaC treatment in a large cohort of patients presenting with primary or recurrent SPSD. This is, to our knowledge, the first substantial cohort reporting quality of life following this technique, with a median follow-up of 33 months. After the initial SiLaC procedure, initial cumulative healing rates were 72.7% for primary SPSD and 70.9% for recurrent SPSD. The remaining patients required at least one more surgical treatment, in most cases a second or third SiLaC. Upon follow-up, approximately 12% of patients in the primary group and 13% of patients in the recurrent group reported persisting symptoms related to SPSD. However, symptom scores at the natal cleft were very low and quality of life at follow-up was significantly better in most SF-36 domains compared with the general USA population.

Several other studies reported outcomes of SiLaC in chronic primary or recurrent SPSD with similar cohort sizes. Pappas et al. [6] reported an initial success rate of 90.3% in 237 patients (210 primary, 27 recurrent), which increased to 97.9% after a second SiLaC treatment. At follow-up (354 days), seven recurrences were observed. Sluckin et al. [7] conducted a multicenter cohort study with 311 patients with primary (*n* = 218, 70.1%) and recurrent disease (*n* = 93, 29.9%), with a follow-up of 10 months. They observed an initial healing rate of 66.2%, increasing to 92.2% and 97.7% after a second and third SiLaC treatment, respectively. Another multicenter retrospective cohort study (*n* = 226) was conducted by de Decker et al., [5] who reported a healing rate of 85.4% after 1–3 SiLaC treatments and a cumulative healing rate (including other treatment modalities) of 93.4%, but at a follow-up of only 129 days. In the current study, the initial healing rate after one SiLaC treatment was 71.9% (72.7% in the primary group, 70.9% in the recurrent group), and after a second, third, or fourth SiLaC treatment, this increased to 86.6% (86.8% in the primary group, 86.4% in the recurrent group), 91.3% (91.4% in the primary group, 91.3% in the recurrent group), and 92.2% (92.2% in both groups) after a second, third, or fourth SiLaC treatment, respectively. Herein, patients treated with incision and drainage for a pilonidal abscess or patients that required another surgical approach after the first, second, or third SiLaC were considered nonhealing. These results are consistent with the results published in the aforementioned studies. However, with a considerably longer follow-up period, the results in this study are, to our opinion, more comprehensive, as they provide a clearer understanding of the mid-term effects of SiLaC.

One study has reported long-term outcomes after SiLaC treatment; Karita et al. [14] reported a reoperation rate of 27.7% (*n* = 23) after a follow-up period of 5.2 years. The reoperation rate is comparable to the one observed in the current study (27.3% in the primary group and 29.1% in the recurrent group). Moreover, Karita et al. reported that recurrent SPSD as an indication for SiLaC was significantly associated with recurrence following SiLaC treatment, compared with SiLaC for primary SPSD. However, such an association was not observed in the current study (*p* = 0.771). This is possibly owing to the difference in definition of recurrent disease; in our study, if a patient underwent surgical incision and drainage of a pilonidal abscess prior to SiLaC treatment, this patient was analyzed in the recurrent group, whereas these patients were analyzed as primary SPSD in the study by Karita et al. It is debatable whether to analyze patients with only previous incision and drainage for a pilonidal abscess in the primary or recurrent group. Since we preferred to keep the primary group as homogeneous as possible, we decided to analyze the group of patients after previous incision and drainage in the recurrent group.

The outcomes of our study align with those reported for other minimally invasive techniques, such as pit-picking and phenolization, particularly when considering the need for repeat procedures, an approach that is common practice in minimally invasive management of SPSD. Gips et al. reported a large cohort study with a mean follow-up of 6.9 years, showing a 5-year recurrence rate of 13.2% after repeat pit-picking, which is lower than the treatment failure rate observed in our study. However, their cohort consisted exclusively of military personnel, which in our opinion introduces substantial selection bias and limits generalizability [1]. Phenolization is likewise frequently used as a staged treatment. Aygen et al. demonstrated that repeat phenolization is a simple and feasible option, reporting recurrence rates of 13.9% after a mean of 3.7 phenol applications per patient with a follow-up of 54.4 months. It should, however, be remarked that these results were based on a relatively small cohort of 36 patients with only recurrent SPSD [17].

To date, no studies have reported quality of life in patients treated with SiLaC. Only a few studies have reported on subjective patients’ satisfaction [8, 18]. These studies reported satisfaction rates of 98.0% and 88.2%, respectively. However, both studies included small patient cohorts, had short follow-up periods, and used nonvalidated questionnaires to report on patients’ satisfaction. The current study is the first to report on quality of life of patients treated with SiLaC using a validated questionnaire, i.e. the short-form 36. In most domains of the SF-36, both treatment groups scored even higher than the general USA population, indicating the positive effect of SiLaC on quality of life in patients with SPSD. This is supported by the finding that over 80% of patients in both groups would choose the same SiLaC treatment again.

Several limitations apply to this study. First, the retrospective design of the study is a limitation. Although the questionnaire was sent prospectively, the retrospective nature of the study makes interpretation of the results prone to bias. For instance, preoperative quality of life scores could not be obtained, which limits the ability to accurately assess changes in postoperative quality of life. For that reason, postoperative quality of life scores from the SF-36 were compared with figures from the general population. However, it should be remarked that this comparison in quality of life between the study population and the general USA population was not adjusted for age. Second, this study lacks a control group. Ideally, SiLaC treatment should be compared with another minimally invasive technique, such as phenolization of the sinus tract or pit-picking alone, preferably in a randomized setting. However, this is challenging as in most hospitals only one kind of minimal invasive technique is available. Although no control group has been included, the mid-term data after SiLaC in a large patient population, as presented in the current study, should be considered as additional value as this is currently very scarce in the literature. Third, we were not able to include all consecutive patients who underwent SiLaC during the inclusion period as those were either not available or not motivated to participate, which might have introduced selection bias.

## Conclusions

SiLaC is a safe procedure with a low complication rate. This study is the first to report on quality of life using a validated questionnaire and with high quality of life scores, the SiLaC procedure appears to be a treatment modality highly appreciated by patients with primary or recurrent SPSD. Mid-term follow-up showed an initial healing rate of 71.9% for the entire cohort after the first SiLaC treatment, increasing after a second, third, or fourth SiLaC treatment, only requiring other surgical treatments in 7.8% of patients with both primary and recurrent SPSD. To further assess the efficacy of SiLaC in comparison to other minimally invasive procedures, additional comparative studies are required.

## Data Availability

No datasets were generated or analyzed during the current study.
